# Fitness and proteome changes accompanying the development of erythromycin resistance in a population of *Escherichia coli* grown in continuous culture

**DOI:** 10.1002/mbo3.121

**Published:** 2013-08-28

**Authors:** Denisa Petráčková, Jiří Janeček, Silvia Bezoušková, Ladislava Kalachová, Zuzana Techniková, Karolína Buriánková, Petr Halada, Kateřina Haladová, Jaroslav Weiser

**Affiliations:** Institute of Microbiology, Academy of Sciences of the Czech RepublicPrague, Czech Republic

**Keywords:** Continuous cultivation system, erythromycin, *Escherichia coli*, fitness, proteome, resistance

## Abstract

We studied the impact of a sublethal concentration of erythromycin on the fitness and proteome of a continuously cultivated population of *Escherichia coli*. The development of resistance to erythromycin in the population was followed over time by the gradient plate method and minimum inhibitory concentration (MIC) measurements. We measured the growth rate, standardized efficiency of synthesis of radiolabeled proteins, and translation accuracy of the system. The proteome changes were followed over time in two parallel experiments that differed in the presence or absence of erythromycin. A comparison of the proteomes at each time point (43, 68, and 103 h) revealed a group of unique proteins differing in expression. From all 35 proteins differing throughout the cultivation, only three were common to more than one time point. In the final population, a significant proportion of upregulated proteins was localized to the outer or inner cytoplasmic membranes or to the periplasmic space. In a population growing for more than 100 generations in the presence of antibiotic, erythromycin-resistant bacterial clones with improved fitness in comparison to early resistant culture predominated. This phenomenon was accompanied by distinct changes in protein expression during a stepwise, population-based development of erythromycin resistance.

## Introduction

The alarming increase in the appearance of antibiotic-resistant bacteria is the result of the increased use of antibiotics, both in humans and in animals, combined with the exceptional ability of bacteria to develop resistance. One proposed strategy to reverse this development is to reduce the use of antibiotics to promote the disappearance of resistant bacteria that are present in human and environmental reservoirs (Chastre 2008). This approach is based on the assumption that resistance is conferred at the cost of impaired survival fitness in the absence of antibiotics compared with sensitive strains (Andersson and Hughes 2010). In general, resistant bacteria seem to be less fit than sensitive strains, which suggests, that resistance may be reversible. An associated question and potential problem is whether the supposedly less fit, more resistant, and very often avirulent bacteria accumulate compensatory mutations that restore fitness and virulence without loss of resistance. This phenomenon would then stabilize the resistant population and allow this population to successfully compete with sensitive organisms, even in an environment depleted of antibiotics (Andersson and Hughes 2010). If compensatory mutations are as common in clinical settings as in the laboratory, many types of resistance will be irreversible (Andersson 2003; Andersson and Hughes 2010).

Macrolides inhibit protein synthesis in a wide range of bacteria by binding to the large ribosomal subunit. Macrolide resistance mechanisms have been characterized for a broad range of Gram-positive and Gram-negative bacteria, including pathogenic isolates and macrolide producers. The most widespread mechanism involves the methylation of an adenine residue within 23S rRNA, conferring resistance to macrolide, lincosamide, and streptogramin B (MLS_B_) antibiotics (Weisblum 1995; Gaynor and Mankin 2003). The ribosomal proteins L4 and L22, which form part of the peptide exit tunnel in the large ribosomal subunit, have long been known to participate in erythromycin resistance mechanisms (Chittum and Champney 1994).

The use of continuous cultures could help to create laboratory conditions that are closer to the conditions found within natural hosts of bacteria, with controlled slow growth rates and a controlled population size. Although the heyday of continuous culture occurred in the 1960s (Malek and Ricica 1966), such culture is again being recognized in the postgenomic era (Hoskisson and Hobbs 2005). This technology was successfully applied in studies of the oral pathogen *Streptococcus mutans*, in which two-dimensional (2D) gel electrophoresis-based proteomic technology was used to identify cellular and extracellular proteins expressed during glucose limitation, which mimics the conditions prevailing in a healthy human oral cavity (Len et al. 2003).

Bacterial fitness represents a complex of physiological parameters, among which translation rate and accuracy play an important role (Lovmar and Ehrenberg 2006). It is particularly surprising how few mutations appear to be required to transform a slow-growing natural isolate with inefficient and inaccurate ribosomes into a growth-optimized laboratory strain (Kurland 1992).

In contrast, it was shown that a single adaptive mutation allowing mutant *Pseudomonas fluorescens* to occupy a niche that its ancestor could not occupy has a wide range of pleiotropic effects on protein levels (Knight et al. 2006). Single base-pair substitution in the coding sequence of a signaling pathway gene in this bacterium led to 52 proteome changes that corresponded to 46 identified proteins, and none of these proteins was required for the adaptive phenotype (Knight et al. 2006).

In this study, we tracked the development of antibiotic resistance and fitness changes in an *Escherichia coli* population cultivated in continuous culture for more than 100 generations in the presence of a sublethal concentration of erythromycin. We measured growth rate, translation rate and accuracy and proteome changes in whole bacterial populations during two parallel experiments that differed in the presence or absence of erythromycin. In the proteome of the bacterial population grown in the presence of erythromycin, which showed a clear tendency toward selection for bacterial clones that were more fit and erythromycin resistant, we identified three groups of unique proteins with expression changes in comparison with a control experiment without antibiotic.

## Materials and Methods

### Bacterial strains and growth media

In all experiments, we used an *E. coli envA* mutant strain (UR172-F′23-5*Δ* [*lac-proB*]*, ara, argE* [*UAG*]*, nalA, rpoB, thi, envA::Tn10, Tet*^*R*^) known to have increased permeability to several antibiotics, including erythromycin (Normark et al. 1969; Andersson and Kurland 1987). To measure nonsense suppression levels, we transformed the strain with the plasmid pLUX2, which bears bacterial luciferase with a UAG stop codon in position 13 of the *luxB* gene (Schultz and Yarus 1990).

Frozen glycerol stock cultures were used to inoculate 25 mL of M9 medium (Pardee et al. 1959) supplemented with 15 μg mL^−1^ chloramphenicol (Amersham LifeScience, England) to maintain pLUX2 and 15 μg mL^−1^ tetracycline (Amersham Life Science, England) to maintain the *envA::Tn10* mutation in the strain. The precultures were grown overnight and used to inoculate the main cultures in glass tube fermenters, in which the cultures were kept for 4–5 h in batch mode before continuous operation was initiated. The medium in the “erythromycin experiment” was supplemented with erythromycin (Amersham Life Science, England) at a concentration of 10 μg mL^−1^. This concentration did not inhibit growth by more than 25%.

### Continuous cultivation

The cultivation vessel of the continuous-culture system was made from a glass tube that was 5 cm in diameter, with a working volume of 50 mL (see Fig. S1). The tube for substrate supply was an integral part of the glass cup of the vessel. Another input port (at the bottom) was for the air supply, and a third port was for the withdrawal of culture broth and air efflux. A multichannel peristaltic pump was used to supply two parallel cultures with medium. A constant volume of culture broth was maintained by the siphon principle. Mixing was achieved via rising air bubbles, and temperature was maintained at 37°C by placing the tubes in a water bath.

### Measurement of erythromycin resistance

The development of erythromycin resistance in the bacterial populations was monitored on gradient agar plates with Luria-Bertani (LB) growth medium and a concentration of erythromycin ranging from 0 to 50 μg mL^−1^. The plates were inoculated with 500 μL of overnight grown sample culture without erythromycin.

Minimal inhibitory concentrations (MICs) were determined by a microdilution assay in sterilized 96-well plates and in a final volume of 100 μL, as follows. Bacteria were grown overnight in LB without erythromycin and diluted in the same medium to reach 10^6^ colony-forming units (CFU) mL^−1^. A 50 μL sample of LB containing bacteria was added to 50 μL of LB containing 0–512 μg mL^−1^ erythromycin (Amersham Life Science, England) in serial twofold dilutions. The inhibition of proliferation was determined by optical density (OD) measurements (600 nm) after an overnight incubation at 37°C.

### 2D polyacrylamide gel electrophoresis and protein visualization

Proteins were precipitated overnight using four volumes of acetone at −20°C. Protein pellets were dissolved in sample buffer containing 7 mol L^−1^ urea, 2 mol L^−1^ thiourea, 4% (w/v) CHAPS (3-[(3-Cholamidopropyl)dimethylammonio]-1-propanesulfonate hydrate), 0.8% Pharmalyte 3–10, 65 mmol L^−1^ dithiothreitol (DTT), and bromophenol blue (Sigma-Aldrich, St. Louis, MO). The samples were soaked into IPG strips (Immobiline DryStrips, 18 cm, pH 3–10 NL (GE Healthcare, Uppsala, Sweden) and rehydrated overnight. In the first dimension, isoelectric focusing (IEF) was performed using voltage that linearly increased to the steady state (the voltage was limited to 150 V for 2 h, 300 V for 2 h, and 3500 V for 5 h) and then stabilized at 3500 V for 31 h (Multiphor II, GE Healthcare/Amersham Pharmacia). After IEF, the strips were washed in equilibration solution (50 mmol L^−1^ Tris-HCl (pH 6.8), 6 mol L^−1^ urea, 30% glycerol, and 2% SDS) containing 0.02 g mL^−1^ DTT for 10 min, followed by a second 10 min wash in equilibration buffer containing 0.025 g mL^−1^ iodoacetamide and a few crystals of bromophenol blue. Sodiumdodecyl sulfate polyacrylamide gel electrophoresis (SDS-PAGE) was performed on 12.5% polyacrylamide slab gels (Investigator; Genomic Solutions, Oxford GlycoSystems, Bedford, MA).

Proteins were visualized by silver staining (ProteoSilver Stain Kit; Sigma-Aldrich) or using colloidal Coomassie Brilliant Blue stain (CBB, Colloidal Blue Staining Kit; Invitrogen, Carlsbad, CA) to obtain reference maps and for mass spectrometry (MS) identification. To obtain autoradiograms, 5 × 10^5^ disintegrations per minute (dpm) were loaded onto each gel. The dried gels with the radioactively labeled proteins were exposed to phosphor screens (Fuji screen, 20 × 25 cm) for 4 days. The gels were scanned at a 100 μm resolution using Molecular Imager FX (Bio-Rad).

### Digitalization of gel images and data quantification

The 2-DE image analysis was performed using PDQuest 7.3.1 software (Bio-Rad, Hercules, CA). For matching and quantification, raw images were smoothed to remove noise, background was subtracted, and spots were detected in uncalibrated quantification mode.

Two biological replicates and three technical replicates were performed. Protein spots showing reproducible changes in protein abundance were considered biomarkers. A statistical analysis was performed using a Student's *t*-test.

### Enzymatic digestion

CBB-stained protein spots were excised from the gel, cut into small pieces, and destained using 50 mmol L^−1^ 4-ethylmorpholine acetate (pH 8.1) in 50% acetonitrile (MeCN). After complete destaining, the gel was washed with water, shrunk by dehydration in MeCN, and reswelled in water. The supernatant was removed and the gel was partly dried in a SpeedVac (Savant, Thermo Scientific, Waltham, MA) concentrator. The gel pieces were then incubated overnight at 37°C in a cleavage buffer containing 25 mmol L^−1^ 4-ethylmorpholine acetate, 5% MeCN, and trypsin (100 ng; Promega, Madison, WI). The resulting peptides were extracted in 40% MeCN/0.1% trifluoroacetic acid (TFA). An aqueous 50% MeCN/0.1% TFA solution of α-cyano-4-hydroxycinnamic acid (5 mg mL^−1^; Sigma) was used as a matrix-assisted laser desorption/ionization (MALDI) matrix. Then, 1 μL of the peptide mixture was deposited on the MALDI plate, allowed to air dry at room temperature and overlaid with 0.4 μL of the matrix.

### MALDI MS and protein identification

Mass spectra were measured on an Ultraflex III MALDI-TOF/TOF (time of flight) instrument (Bruker Daltonics, Bremen, Germany) in the mass range of 700–4000 Da and calibrated internally using the monoisotopic [M+H]^+^ ions of trypsin auto-proteolytic fragments (842.5 and 2211.1 Da). The peak lists created using the flexAnalysis 3.0 program were searched using an in-house MASCOT (Matrix Science Inc, Boston, MA) search engine against the SwissProt 2011_12 database subset of *E. coli* proteins, with the following search settings: peptide tolerance of 20 ppm, missed cleavage site value set to two, variable carbamidomethylation of cysteine, oxidation of methionine, and protein N-terminal acetylation. Proteins with a molecular weight search (MOWSE) score, calculated for the settings used, exceeding the threshold of 56 were considered to be identified. If the score was lower or only slightly higher than the threshold value, the identity of the protein candidate was confirmed by MS/MS analysis.

## Results

In an experiment encompassing the continuous cultivation of two parallel populations of the *E. coli envA* mutant strain, one in the presence of 10 μg mL^−1^ erythromycin (erythromycin population) and the other in the absence of antibiotic (control), we tracked the growth rate of the bacterial populations, which is an accepted measure of fitness. The cultures were maintained at a volume of 50 mL in small glass fermenters by regulating the inflow of fresh medium and keeping the OD of the cultures at a value of 1.2 (see Fig. S1). The OD of the cultures was measured in 2–10 h intervals, and samples were collected after 0, 43, 68, and 103 h of cultivation for proteome analysis. For each sample, we measured fitness, antibiotic resistance, and translation accuracy.

### Fitness of cultures

**Figure 1:**
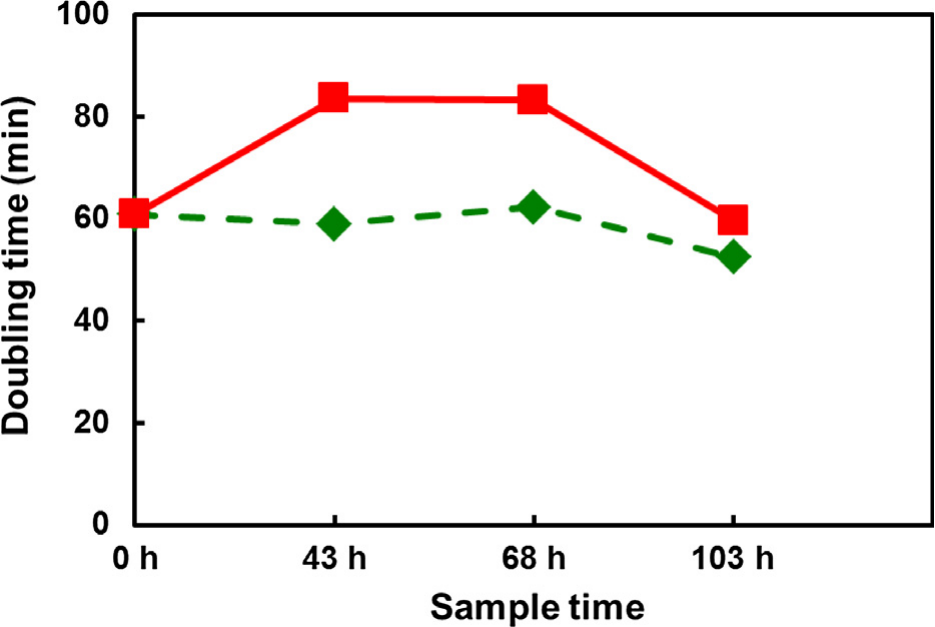
Comparison of changes in doubling time for *Escherichia coli* cultures continuously grown in the absence (-◊-) or presence (-□-) of erythromycin (10 μg mL^−1^).

Culture fitness, which was measured by doubling time, was virtually unchanged in the control population without antibiotic for more than 70 generations (68 h), and the generation time remained at 60 min. Later (103 h) the culture fitness of the control population even improved, and the generation time shortened to 50 min (Fig. 1). In the erythromycin containing population, the culture soon became less fit, which was indicated by an increase in the generation time of more than 25%, corresponding to a value of 83 min. The culture remained at this level until 68 h of cultivation (∼50 generations), and then the culture fitness significantly increased, reaching a generation time of 60 min at 103 h of cultivation (more than 80 generations), which was equal to the generation time at the start of the experiment (Fig. 1).

### Erythromycin resistance

The development of erythromycin resistance in the continuously grown bacterial populations was followed over time by growing the cultures on erythromycin-gradient agar plates (0–50 μg mL^−1^) and defining the MIC values of the populations in the liquid cultures. In the control culture, the resistance to erythromycin on agar plates did not change throughout the entire experiment (Fig. 2, upper panel). The resistance to erythromycin in the culture continuously grown in the presence of erythromycin (10 μg mL^−1^) increased over the course of cultivation (Fig. 2, lower panel). At 68 h, we did observe fewer than 100 clones of *E. coli* with higher resistance than the control. At 103 h of cultivation, we counted hundreds of clones with increased resistance to erythromycin on the agar plates.

**Figure 2:**
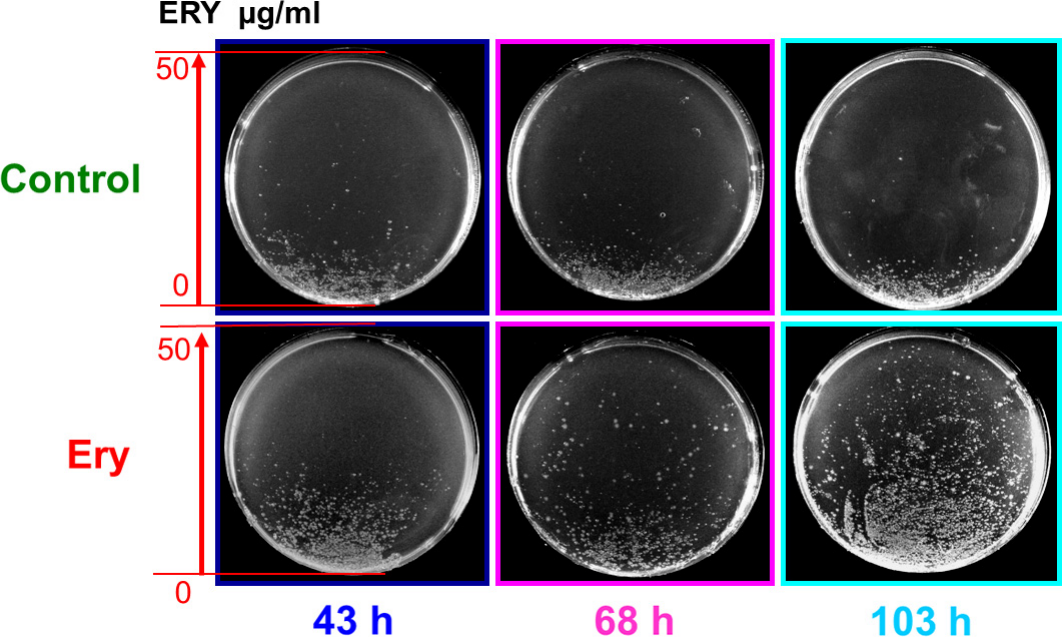
Erythromycin (0–50 μg mL^−1^)-gradient plates showing the development of erythromycin resistance in *Escherichia coli* cultures continuously grown in the presence of the antibiotic (10 μg mL^−1^). The upper line of plates (Control) shows the samples withdrawn from the culture without antibiotic at 43, 68, and 103 h. The lower line (Ery) shows the samples withdrawn from the culture grown in the presence of erythromycin.

The MIC values measured by the microdilution method at 0, 50, 100, and 120 min confirmed an increase and step-like changes in the levels of resistance in the erythromycin culture by a factor of two (see Table S1).

### Analysis of proteomes in erythromycin and control cultures

Comparisons of gels with separated radiolabeled proteins from the erythromycin and control cultures revealed a total of 61 spots with different densities (different by a factor of two or higher). All of the proteins present in the selected spots were identified by MS (see Table S2). From this group of 91 proteins, we eliminated those proteins that were not unique within the spot or conclusively identified. As a result, we identified 35 proteins over the course of the experiment. The proteins identified in samples at individual time points were unique to each time point, except for three (see Tables 1–3). Three time-point samples (43, 68, and 103 h) for which we analyzed the radiolabeled proteomes showed remarkable differences in the number and expression of identified proteins. In the erythromycin culture, at 43 h, we identified 14 up- and three downregulated proteins. At 103 h, there were also 14 upregulated proteins and only one downregulated protein. Surprisingly, in the bacterial population at 68 h, we identified only six downregulated proteins (see Figs. 5). This “switch-like” change in gene expression was more distinctly noticeable when we analyzed the raw gel data (all differing spots without identification). The data were normalized by calculating the ratio of the density of each spot to the density (expression value) of the spot(s) representing the protein synthesis elongation factor Tu, an internal standard in the analyzed gel sets. Here, in comparing the erythromycin culture to the control, we observed the “expression switch” effect, as most differing proteins were first downregulated at 43 h and then, via an expression equilibrium at 68 h, upregulated in the final population at 103 h (see Fig. S2).

**Table 1:** Functional identification of proteins differing from the control in the 43 h sample of the *Escherichia coli* culture continuously grown in the presence of erythromycin (10 μg mL^−1^)

No.	Protein name	Gene	Function	43 h	68 h	103 h
7202	Dihydrodipicolinate synthase	*dapA*	Amino acid metabolism	Up		
7610	Anthranilate synthase component II	*trpD*	Amino acid metabolism	Up		
7401	Carbamoyl-phosphate synthase small chain	*carA*	Amino acid metabolism	Up		
8201	Succinyl-CoA ligase [ADP-forming] subunit alpha	*sucD*	Carbohydrate metabolism	Up		
2901	Aconitate hydratase 2	*acnB*	Carbohydrate metabolism	Up		
4202	Glyoxylate/hydroxypyruvate reductase B	*ghrA*	Carbohydrate metabolism	Up		
6603	Pyruvate kinase I	*pykF*	Carbohydrate metabolism	Up		
8205	Glyceraldehyde-3-phosphate dehydrogenase A	*gapA*	Carbohydrate metabolism	Up		
7609	Fumarate hydratase class I, aerobic	*fumA*	Carbohydrate metabolism	Up		
0002	Acyl carrier protein	*acpP*	Lipid metabolism	Up		Up
8302	HTH-type transcriptional repressor purR	*purR*	Transcription factors	Up		
1406	DNA-directed RNA polymerase subunit alpha	*rpoA*	Transcription	Up		
7507	Bifunctional protein glmU	*glmU*	Cell wall metabolism	Up		
8202	Tryptophanyl-tRNA synthetase	*trpS*	Translation	Up		
0004	Type-1 fimbrial protein, A chain	*fimA*	Cell adhesion	Down	Down	
5103	Outer membrane protein A	*ompA*	Outer membrane proteins	Down		
6201	Methionine aminopeptidase	*map*	Proteolysis	Down		

The proteins' up- or downregulation is marked for each sampling time point.

**Table 2:** Functional identification of proteins differing from the control taken from the 68 h sample of the *Escherichia coli* culture continuously grown in the presence of erythromycin (10 μg mL^−1^)

No.	Protein name	Gene	Function	43 h	68 h	103 h
0004/0009	Type-1 fimbrial protein, A chain	*fimA*	Cell adhesion	Down	Down	
0204/0202	Spermidine/putrescine-binding periplasmic protein	*potD*	Polyamine transport		Down	Up
2003	Universal stress protein A	*uspA*	Stress response		Down	
2004	10 kDa chaperonin	*groL*	Chaperon		Down	
3006	30S ribosomal protein S6	*rpsF*	Translation		Down	
8402	Nicotinate phosphoribosyltransferase	*pncB*	Nucleotide metabolism		Down	

The proteins' up- or downregulation is marked for each sampling time point.

**Table 3:** Functional identification of proteins differing from the control taken from the 103 h sample of the *Escherichia coli* culture continuously grown in the presence of erythromycin (10 μg mL^−1^)

No.	Protein name	Gene	Function	43 h	68 h	103 h
5201	2,3,4,5-tetrahydropyridine-2,6-dicarboxylate N-succinyltransferase	*dapD*	Amino acid metabolism			Up
2101	Histidine-binding periplasmic protein	*hisJ*	Amino acid transport			Up
0005	Glucose-specific phosphotransferase enzyme IIA component	*ptsG*	Sugar transport			Up
5206	UTP–glucose-1-phosphate uridylyltransferase	*galU*	Carbohydrate metabolism			Up
0002	Acyl carrier protein	*acpP*	Lipid metabolism	Up		Up
1201	Malonyl CoA-acyl carrier protein transacylase	*fabD*	Lipid metabolism			Up
5003	ATP-dependent Clp protease proteolytic subunit	*clpA*	Proteolysis			Up
1104	Agmatinase	*speB*	Polyamine synthesis			Up
0204	Spermidine/putrescine-binding periplasmic protein	*potD*	Polyamine transport		Down	Up
5106	NADP-dependent L-serine/L-allo-threonine dehydrogenase ydfG	*ydfG*	E-Oxidoreductases			Up
0101	Fe/S biogenesis protein nfuA	*nfuA*	Stress response			Up
1105	MltA-interacting protein	*mipA*	Outer membrane proteins			Up
0301	Outer membrane protein C	*ompC*	Outer membrane proteins			Up
0201	Uncharacterized protein yceD	*yceD*	Uncharacterized protein			Up
7603	ATP synthase subunit alpha	*atpA*	ATP synthesis			Down

The proteins' up- or downregulation is marked for each sampling time point.

**Figure 3:**
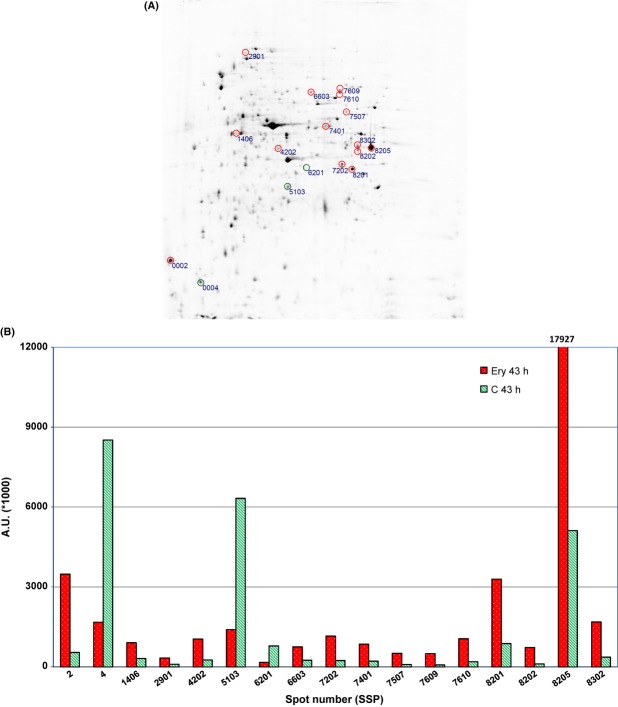
(A) 2-DE proteome map from a 43 h sample of an *Escherichia coli* culture continuously grown in the presence of erythromycin (10 μg mL^−1^). The gels with proteins labeled with ^35^S methionine were exposed to phosphor screens for 4 days and scanned at a 100 μm resolution using a Molecular Imager FX (Bio-Rad). The proteins with expression levels that differ from the control levels are marked with red circles and spot numbers and are listed in Table 1. (B) Graphical presentation showing differences in protein expression in arbitrary units, representing the densities of spots for selected proteins from the 43 h samples of *E. coli* cultures continuously grown in the absence (green) or presence (red) of erythromycin (10 μg mL^−1^). The spot numbers correspond to the spots marked on the 2-DE gel (A).

**Figure 4:**
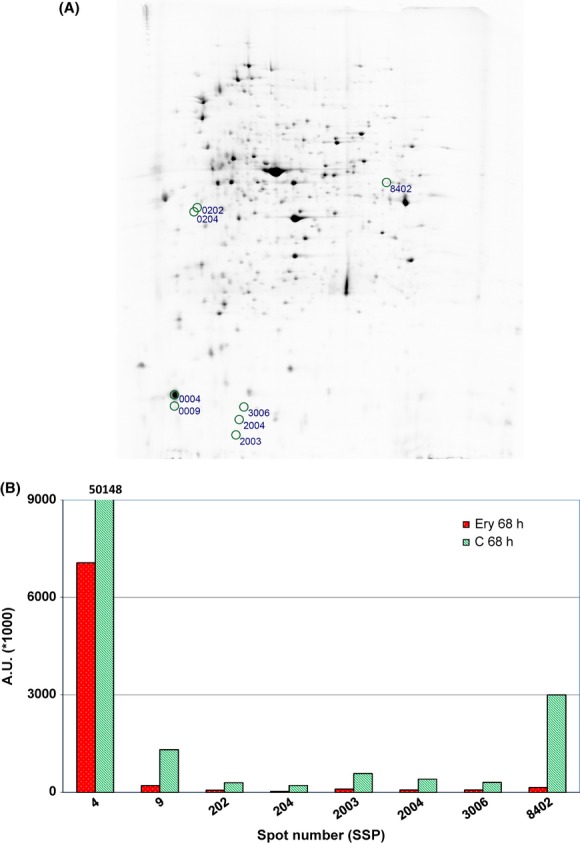
(A) 2-DE proteome map from the 68 h sample of an *Escherichia coli* culture continuously grown in the presence of erythromycin (10 μg mL^−1^). The gels with proteins labeled with ^35^S methionine were exposed to phosphor screens for 4 days and scanned at a 100 μm resolution using Molecular Imager FX (Bio-Rad). The proteins with expression levels that differ from the control are marked with red circles and spot numbers and are listed in Table 2. (B) Graphical presentation showing differences in protein expression in arbitrary units representing the densities of spots for selected proteins from the 68 h samples of *E. coli* cultures continuously grown in the absence (green) or presence (red) of erythromycin (10 μg mL^−1^). The spot numbers correspond to those marked on the 2-DE gel (A).

**Figure 5:**
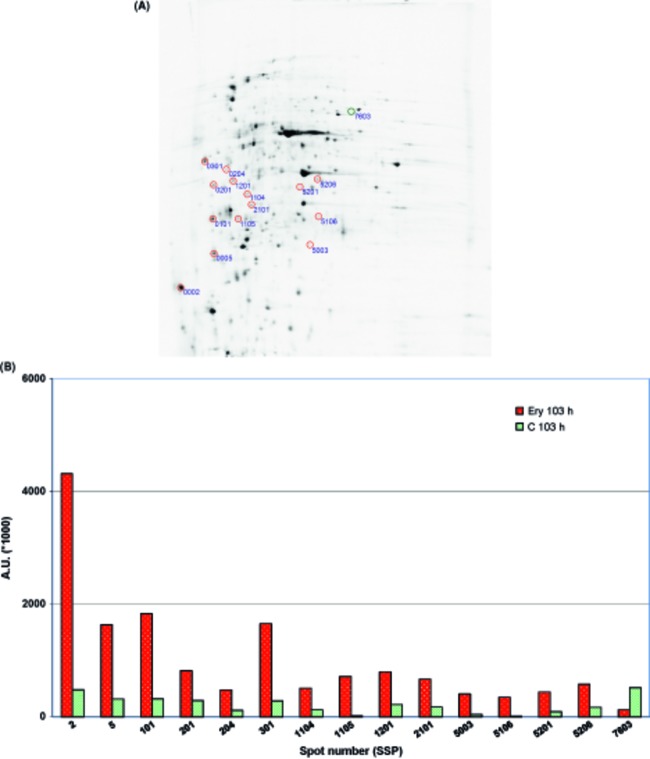
(A) 2-DE proteome map from a 103 h sample of *Escherichia coli* culture continuously grown in the presence of erythromycin (10 μg mL^−1^). The gels with proteins labeled with ^35^S methionine were exposed to phosphor screens for 4 days and scanned at a 100 μm resolution using a Molecular Imager FX (Bio-Rad). The proteins with expression levels that differ from the control levels are marked with red circles and spot numbers and are listed in Table 3. The LuxA subunit of the luciferase reporter system is marked with a blue circle. (B) Graphical presentation showing differences in protein expression in arbitrary units, representing the densities of spots for selected proteins from the 103 h samples of *E. coli* cultures continuously grown in the absence (green) or presence (red) of erythromycin (10 μg mL^−1^). The spot numbers correspond to the spots marked on the 2-DE gel (A).

### Identification of proteins

In the group of 14 upregulated proteins at 43 h, a major proportion of the proteins belonged to pathways for carbohydrate metabolism (six proteins) and amino acid metabolism (three proteins), and there was one protein representing lipid metabolism (acyl carrier protein). This protein was also upregulated in the population at 103 h of cultivation. The four remaining proteins comprised two that were involved in transcription (the alpha subunit of RNA polymerase and PurR repressor), one protein (GlmU) involved in the acetyl transfer step of cell wall synthesis and one tRNA synthetase (TrpS). We identified only three downregulated proteins at 43 h: fimbrial protein (FimA), a part of the pilus structure, which was also downregulated at 68 h; OmpA porin; and methionine aminopeptidase (MAP), which is involved in protein maturation (see Fig. 3 and Table 1).

In the populations at 68 h, there were only a few differences between the proteomes from the erythromycin and control cultures. All six proteins identified at that time point were downregulated in the erythromycin population. Of these proteins, FimA was also downregulated in the 43 h population. Conversely, PotD, which is involved in polyamine transport, was upregulated in the 103 h population. Two proteins were involved in the stress response (UspA and GroL), and the last two identified proteins were the ribosomal protein S6 and PncB, which are involved in the control of nicotinamide adenine dinucleotide (NAD) cofactor synthesis (see Fig. 4 and Table 2).

**Figure 6:**
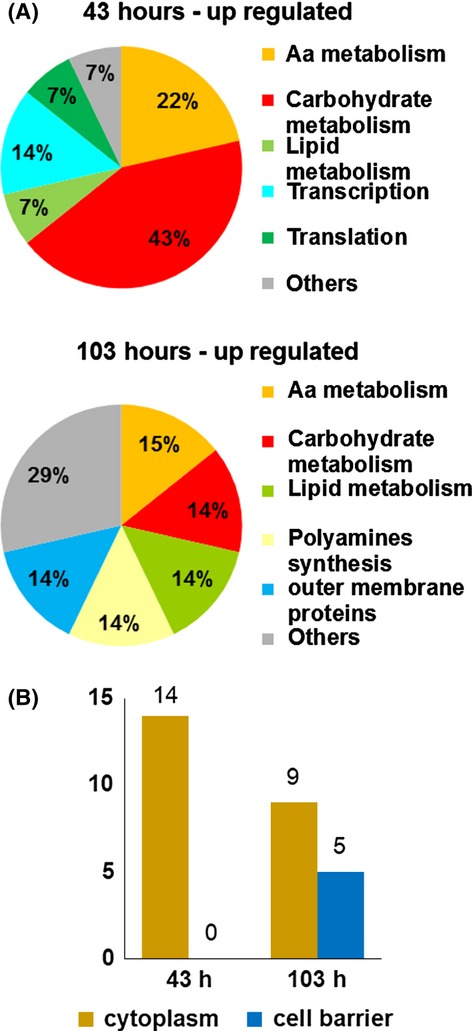
(A) Functional classification of the proteins identified in the 43 h and 103 h samples from *Escherichia coli* cultures continuously grown in the absence or presence of erythromycin (10 μg mL^−1^) that differ in expression. (B) Cellular distribution of proteins (cytoplasmic/cell barrier) identified in the 43 h and 103 h samples from *E. coli* cultures continuously grown in the absence or presence of erythromycin (10 μg mL^−1^) that differ in expression.

The proteins identified in the populations at 103 h of cultivation formed a much less well-ordered group. In the erythromycin culture, which was characterized by improved fitness and antibiotic resistance, 14 proteins were upregulated, and one protein (an ATP synthase subunit) was downregulated. Among the upregulated proteins, there were two representatives each of amino acid metabolism (DapD and HisJ), carbohydrate metabolism (PtsG and GalU), lipid metabolism (AcpP and FabD), and polyamine metabolism (SpeB and PotD). From the remaining proteins, it is worth mentioning the upregulation of the porin OmpC and ClpA protease (see Fig. 5 and Table 3). The functional classification of the proteins identified in the 43 h and 103 h samples is summarized in Figure 6A. We noticed one interesting common feature in comparing these two samples: a high proportion of proteins found in cell barrier structures, represented by the outer or inner membranes. In these structures, five of 14 upregulated proteins were present at 103 h, whereas at 43 h, all 14 upregulated proteins were found in the cytoplasm (see Fig. 6B).

### Translation rate and accuracy

**Figure 7:**
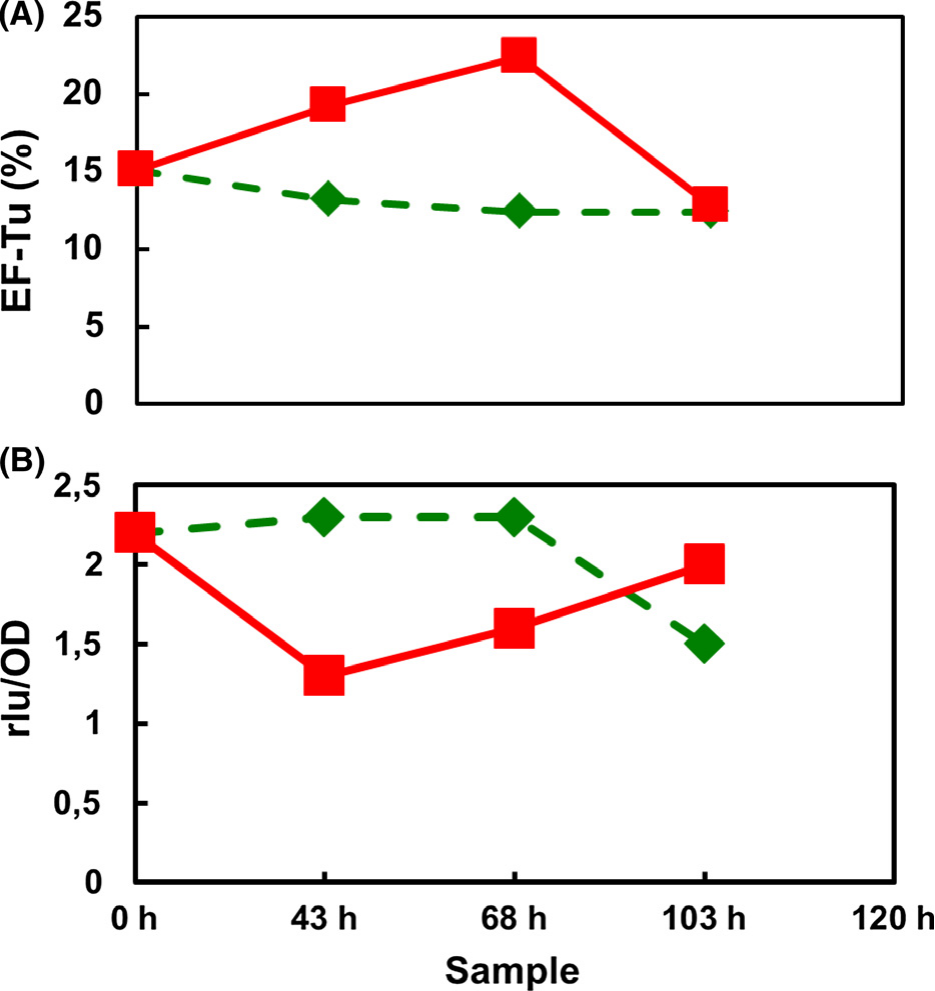
(A) Comparison of changes in the EF-Tu concentration in *Escherichia coli* cultures continuously grown in the absence (-◊-) or presence (-□-) of erythromycin (10 μg mL^−1^). (B) Comparison of changes in translation accuracy for *E. coli* cultures continuously grown in the absence (-◊-) or presence (-□-) of erythromycin (10 μg mL^−1^). Accuracy is represented as the ratio of relative light units to the culture OD. The light units are generated by the active luciferase enzyme when a stop codon inserted into the proximal portion of the B subunit is accidentally read as a sense codon.

As another measure of the fitness of the studied bacterial populations, we calculated the ratio of synthesized EF-Tu to the sum of all synthesized proteins from the radioactive 2D gel data. This value gives information about the amount of EF-Tu that must be synthesized for the production of a given amount of protein in the analyzed population. This value also provides information about the effectiveness of the translation system. Although this parameter did not change for the control culture, in the erythromycin culture, the ratio increased by more than 50% in the 43 h population and by 80% in the 68 h population. In the population at 103 h, which was characterized by increased fitness and erythromycin resistance, protein synthesis occurred with the same amount of EF-Tu as in the control population (see Fig. 7A).

Using a bacterial luciferase reporter gene system, we measured the nonsense suppression rate of an inserted UAG stop codon in selected populations. Measured values (relative light units) are proportional to the number of times that the stop codon is read as a sense codon. In other words, more light indicates less accuracy. In the control culture, this parameter decreased in the 103 h population by 30%; the culture thus became more “accurate.” In the erythromycin culture, the highest level of “accuracy” was reached at 43 h; however, the final population (103 h) was much less accurate, nearly reaching the initial value for nonsense suppression (see Fig. 7B). The presence of the reporter system was confirmed by the identification of the synthesized alpha subunit of luciferase in the 103 h sample (spot 1503/LuxA in Fig. 5).

## Discussion

In this study, we tracked the physiological and proteome changes of *E. coli* susceptible to erythromycin during continuous cultivation in the presence of a sublethal concentration of the antibiotic. In comparison with the control culture, which was grown in parallel without antibiotic, we observed significant changes in the fitness and erythromycin resistance of the erythromycin culture, which was grown for more than 100 generations. As a result of antibiotic pressure, we obtained a population of bacteria that was growing nearly as fast as the population at the start of the experiment but was more resistant to erythromycin. Radiolabeling of the proteins allowed us to identify the proteins that were synthesized during the labeling period (60 min) and to determine the overall efficiency of the translation system. The length of the labeling period was very close to the doubling time of a culture growing at steady state, so we could analyze gene expression throughout the cell cycle reasonably well.

During the early stages in which the cells had to cope with the metabolic burden of the presence of an antibiotic, there was a prevalence of the upregulation of proteins involved in energy and general housekeeping metabolism, whereas in the final population, much fewer of these proteins were upregulated.

When we compared protein expression in the erythromycin-treated culture with the development of translation accuracy, we could see two distinct culture states and one transient period (68 h) logically joining the states (see Fig. S2). At the beginning (43 h), the culture was coping with the pressure of the antibiotic, which inhibits protein synthesis. This phenomenon resulted in slower growth and the general downregulation of protein synthesis at the expense of the rather high accuracy of the process. This development was followed by a transient period, reflected only by a few changes in the expression of proteins in comparison with the control. This culture was still slow growing and translation was occurring with higher accuracy than in the control. The final population, which passed through more than 100 generations, was generally characterized by the upregulation of all proteins differing from the control, and the culture grew faster than at the beginning of cultivation, whereas the accuracy of the translation system was lower.

By analyzing EF-Tu-normalized protein expression, we could look at one part of translation system efficiency, which was affected in the initial culture by erythromycin, which most likely easily entered the cells. This event apparently led to slower growth with higher translation accuracy.

In the final culture, there were most likely already available mechanisms protecting the translation system against the antibiotic, either by affecting the level of erythromycin transport into the cell or by protecting the ribosomes, which are the primary targets of the drug, via certain stable mutations in the rRNA or ribosomal proteins. Strong candidates for such modifications are the L4 and L22 ribosomal proteins and 23S rRNA (Mankin 2008). Erythromycin, which belongs to a group of hydrophobic antibiotics, gains access to the cell interior by permeating the outer membrane bilayer. In a large number of bacterial species, it has been shown that drug-resistant strains possess modifications in the lipid or protein composition of the outer membrane (Delcour 2009).

When we examined the compartmentalization of upregulated proteins in a young bacterial population (43 h) cultivated in the presence of erythromycin, all of the proteins (14) were present in the cytoplasm. In contrast, in the final population (103 h), which had increased resistance and higher fitness, five of 14 proteins were present in the outer or inner membrane or in the periplasmic space (see Fig. 6B).

It is also noteworthy that in the 103 h population, we observed the upregulation of the OmpC porin together with two proteins involved in polyamine synthesis and transport (SpeB and PotD) because in *E. coli*, OmpF and OmpC are inhibited by the polyamines spermine, spermidine, and cadaverine (Iyer and Delcour 1997; Delcour 2009).

The role of polyamine metabolism enzymes, and polyamines in particular, might also be in the regulation of growth rate because DNA microarray assays for *E. coli* showed that polyamines are able to upregulate 309 genes (Igarashi and Kashiwagi 2006). This upregulation is very often achieved via increasing the levels of transcription factors, creating a so-called polyamine modulon (Igarashi and Kashiwagi 2006). We observed the downregulation of PotD in the slow-growing population at 68 h and upregulation in the fast-growing final (103 h) population in the erythromycin culture. Another interesting role of polyamines was discovered in the study of the effect of gaseous ammonia on modification of antibiotic resistance in several Gram-negative and Gram-positive bacteria. In *E. coli* the exposure to ammonia increased the level of intracellular polyamines, which led to modification in membrane permeability to different antibiotics (Bernier et al. 2011).

In the final population of bacteria grown in the presence of erythromycin, it is likely that not all bacteria equally contributed to the observed increased resistance, as demonstrated by the distribution of clones with different erythromycin resistance on antibiotic-gradient plates (Fig. 2). Additionally, the accompanying protein expression changes in the proteomes of the cultures were not continuous but rather of a discrete nature (“expression switch”). This phenomenon resembles the mechanism of the induction of population-based antibiotic resistance via indole signaling, which activates drug efflux pumps and protective mechanisms against oxidative stress (Lee et al. 2010). In this way, a few highly resistant mutants improve the survival of the less resistant constituents of the population.

By coincidence, in our cultivation system (both the control and the erythromycin cultures), we used relatively high concentrations of chloramphenicol (15 μg mL^−1^) to maintain the luciferase plasmid in the cells and tetracycline (15 μg mL^−1^) to retain the transposon-mediated *envA* mutation. These drugs have long been known to be potent inhibitors of indole synthesis (Gibson et al. 1956). The concentrations we used were far higher than the levels found to inhibit indole synthesis in vivo (Gibson et al. 1956), and we did not see any upregulation of the *tnaA* gene, which codes for tryptophanase-producing indole (Lee et al. 2010). This finding suggests that here, we might also be seeing a population-based antibiotic resistance system generating antibiotic resistance in bacteria cultivated for a long time with erythromycin but most likely without indole signaling. The evolution of a similar stepwise resistance mechanism was recently described in the case of the antibiotic trimethoprim through an emergence of mutations restricted to the gene encoding the enzyme dihydrofolate reductase (Toprak et al. 2012).

Our results support the hypothesis that the long-term application of selective antibiotic pressure can generate an antibiotic-resistant bacterial subpopulation for which the handicap of being less fit is eliminated by the accumulation of adaptive changes (mutations) that return the fitness of the population to the original value.

## Supporting Information

**Figure S1.** Continuous cultivation equipment set up with two glass fermenters.

**Figure S2.** Graphical presentation of protein expression differences for all PDQuest software selected protein spots from 43, 68 and 103 h samples of *E. coli* culture continuously grown in absence (green) and presence (red) of erythromycin (10 μg/mL). The spot density values in arbitrary units are normalized to EF-Tu concentration as a measure of the overall translation system efficiency.

**Table S1.** Erythromycin resistance represented by MIC values for 0, 50, 103, and 120 h samples of *E. coli* culture continuously grown in the absence (Control) and presence (Ery) of erythromycin (10 μg/mL). MIC values were determined by microdilution assay performed in 96-well plates in a final volume of 100 μL. Inhibition of proliferation was determined by optical density measurements (600 nm) after overnight incubation at 37°C.

**Table S2.** Complete list of MALDI functionally classified proteins in all the spots differentially selected by PDQuest software from comparison of 2-DE proteomes from erythromycin and control experiment.
