# Supplementary figures and images for: Fitness and proteome changes accompanying the development of erythromycin resistance in a population of Escherichia coli grown in continuous culture
Source: Microbiologyopen. 2013 Aug 28;2(5):841–52. doi: 10.1002/mbo3.121 (PMC3831644; doi:10.1002/mbo3.121)

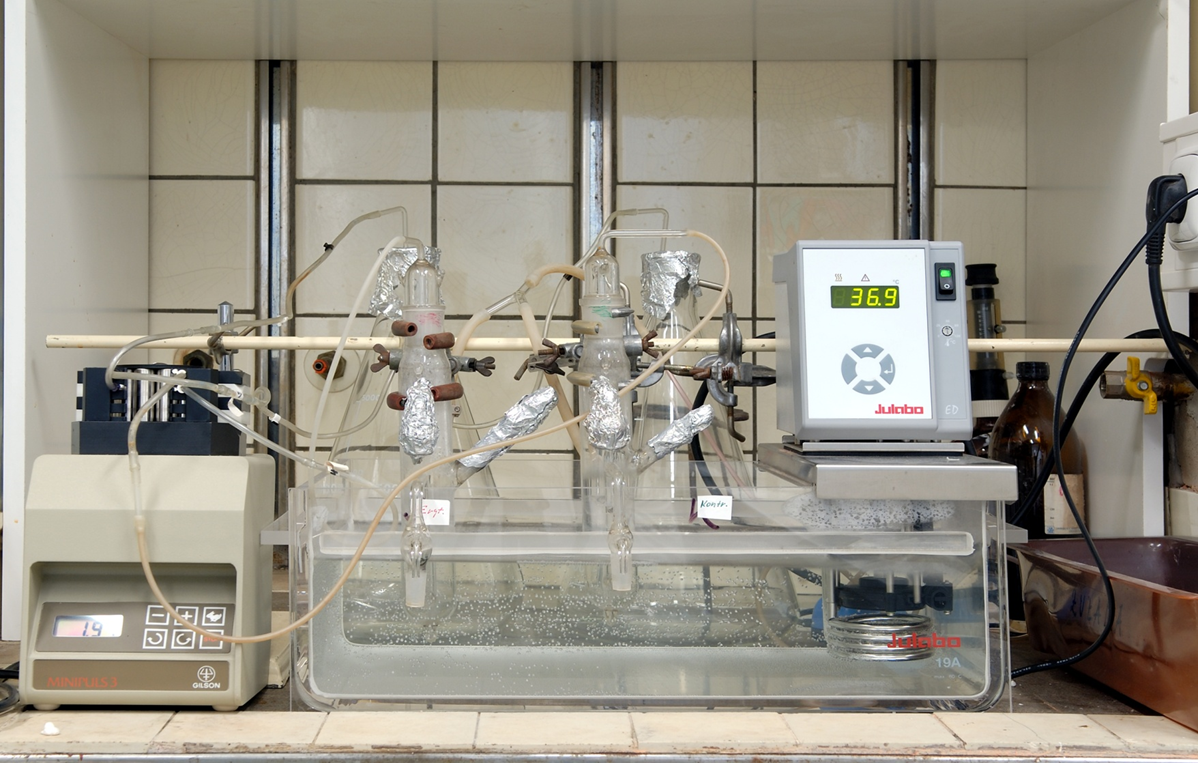

Supplement: Supplementary file 1 [file mbo30002-0841-SD1.tif]
