# Supplementary material for: Fitness and proteome changes accompanying the development of erythromycin resistance in a population of Escherichia coli grown in continuous culture
Source: Microbiologyopen. 2013 Aug 28;2(5):841–52. doi: 10.1002/mbo3.121 (PMC3831644; doi:10.1002/mbo3.121)
Supplement: Supplementary file 4 [file mbo30002-0841-SD4.pdf]

Spot No	Protein name	DTB No.	No. peptides	Coverage [%]	MS/MS confirmation	MW	pI
0002	Acyl carrier protein	ACP_ECOLI	2(3)	32(34)	ITTVQAAIDYINGHQA STIEERVKKIIGEQLGVKQ	9	4.0
0004	Type-1 fimbrial protein, A chain	FIMA1_ECOLI	5	60	AATTVNGGTVHFK YFATGAATPGAANADATFK GEVVNAACAVDAGSVDQTVQ LGQVR TGAALTLDGATFSSETTLNNG TNTIPFQAR	18	5.1
0005	Glucose-specific phosphotransferase enzyme IIA component	PTGA_ECOL6	9	59	No	18	4.7
0009	Type-1 fimbrial protein, A chain	FIMA1_ECOLI	4	50	No	18	5.1
0101	Fe/S biogenesis protein nfuA	NFUA_ECOLI	5	26	LLANQEEGTQIR VEYMLQSQINPQLAGHGGR	21	4.5
0104	Adenylyltransferase thiF	THIF_ECOLI	6	29	LLSGIETPAGELR LTQLNPDIQLTALQQR	27	4.7
0104	FKBP-type peptidyl-prolyl cis-trans isomerase slyD	SLYD_ECO57	5	28	FNVEVVAIR DLVVSLAYQVR	21	4.9
0201	Uncharacterized protein yceD	YCED_ECO57	6	26	LDYQGIYTPDQVER RLDYQGIYTPDQVER	19	4.5

0204	Spermidine/putrescine-binding periplasmic protein	POTD_ECOLI	10	36	LINFLLRPDVAK	39	5.2
0301	Outer membrane protein C	OMPC_ECOLI	19	68	No	40	4.6
1009	Putative peroxiredoxin bcp	BCP_ECO57	8	51	VLVYFYPK FSLPDQDGEQVNLTDFQGQR	18	5.0
1009	Deoxyuridine 5'-triphosphate nucleotidohydrolase	DUT_ECO24	5	43	GQDSFTIQPGER EFPLPTYATSGSAGLDLR	16	4.9
1010	NifU-like protein	NIFU_ECOLI	4	41	VIDHYENPR VNDEGIIEDAR	14	4.8
1104	Agmatinase	SPEB_ECOLI	11	48	No	34	5.1
1105	MltA-interacting protein	MIPA_ECOLI	12	76	FTQYGYLR TTLAGDTLDNSNGIVWDMAW LYR	28	5.5
1201	Malonyl CoA-acyl carrier protein transacylase	FABD_ECOL6	14	67	No	32	5.0
1406	DNA-directed RNA polymerase subunit alpha	RPOA_ECOLI	16	53	No	37	5.0
1503	Alkanal monooxygenase alpha chain	LUXA_VIBHA	18	52	LFQSDVMPYLK VFGTDMDNSR IDHCLSYITSVDHDSNR	40	5.0
2003	Universal stress protein A	USPA_ECOLI	6	41	QLINTVHVDMLIVPLRDEEE	16	5.1

2004	10 kDa chaperonin	CH10_ECOLI	5	47	VGDIVIFNDGYGVK	10	5.2
2101	Histidine-binding periplasmic protein	HISJ_ECOLI	8	39	No	28	5.5
2107	Transcriptional regulatory protein phoP	PHOP_ECO57	11	48	NSGLASQVISLPPFQVDLSR GQGYLFELR	25	5.0
2107	2-keto-4-pentenoate hydratase	MHPD_ECOLI	6	33	GSECLGHPLNAAVWLAR DLIGIDNAEAAYAIQHINVQHD VAQGR	29	5.1
2107	Aerobic respiration control protein arcA	ARCA_ECO57	7	33	FNGWELDINSR HFESTPDTPEIIATIHGEGYR	27	5.2
2302	UTP--glucose-1-phosphate uridylyltransferase	GALU_ECOLI	12	55	No	33	5.1
2504	ATP-dependent Clp protease ATP-binding subunit clpX	CLPX_ECO24	11	31	FGLIPEFIGR LIEGTVAAVPPQGGR	46	5.2
2504	Isocitrate dehydrogenase [NADP]	IDH_ECOLI	6	19	STQVYGQDVWLPAETLDLIR HPELTDMVIFR DWGYQLAR	46	5.2
2605	Asparaginyl-tRNA synthetase	SYN_ECOLI	16	39	No	53	5.2
2901	Aconitate hydratase 2	ACON2_ECOLI	18	21	No	93	5.2
3006	30S ribosomal protein S6	RS6_ECOLI	7	49	NVEAPQEVIDELETTFR FNDAVIR AHYVLMNVEAPQEVIDELETT FR	16	4.9

3007	30S ribosomal protein S6	RS6_ECO27	9	65	NVEAPQEVIDELETTFR HYEIVFMVHPDQSEQVPGMIER	15	5.3
3007	50S ribosomal protein L17	RL17_ECOLI	3	24	AGDNAPMAYIELVDR LFNELGPR	14	11.1
3504	Enolase	ENO_ECO24	13	47	No	46	5.3
3504	Elongation factor Tu 1	EFTU1_ECO24	5	19	GITINTSHVEYDTPTR ELLSQYDFPGDDTPIVR	43	5.3
4105	Protease 7	OMPT_ECOLI	12	40	GWLLNEPNYR VYVEGAWNR	36	5.8
4105	Outer membrane protein A	OMPA_ECOLI	6	23	LGYPITDDLDIYTR NHDTGVSPVFAGGVEYAITPEIATR	37	6.0
4105	Beta-lactamase TEM	BLAT_ECOLX	19	63	No	31	5.7
4202	Glyoxylate/hydroxypyruvate reductase B	GHRB_ECOLI	10	34	No	35	5.5
4206	Spermidine synthase	SPEE_ECOLI	10	55	No	32	5.3
4303	Aspartate--ammonia ligase	ASNA_ECOLI	12	46	No	37	5.5
4604	Dihydrolipoyllysine-residue succinyltransferase component of 2-oxoglutarate dehydrogenase complex	ODO2_ECOLI	16	45	No	44	5.6
4605	Alkyl hydroperoxide reductase subunit F	AHPF_ECOLI	16	39	ASLSAFDYLIR KPSFLITNPGSNQGPR	56	5.5

4605	Glucose-6-phosphate 1-dehydrogenase	G6PD_ECOLI	10	27	WAGVPFYLR FANSLFVNNWDNR	56	5.6
5003	ATP-dependent Clp protease proteolytic subunit	CLPP_ECOLI□	7	36	VMIHQPLGGYQGQATDIEIHAR ALVPMVIEQTSR	23	5.5
5103	Outer membrane protein A	OMPA_ECOLI	12	44	No	37	6.0
5106	NADP-dependent L-serine/L-allo-threonine dehydrogenase ydfG	YDFG_ECOLI	8	45	No	27	5.7
5201	2,3,4,5-tetrahydropyridine-2,6-dicarboxylate N-succinyltransferase	DAPD_ECOLI	13	63	No	30	5.6
5206	UTP--glucose-1-phosphate uridylyltransferase	GALF_ECOLI	10	49	No	33	5.7
5504	Xaa-Pro dipeptidase	PEPQ_ECOLI	16	42	No	50	5.6
6103	Chloramphenicol acetyltransferase	CAT_ECOLX	9	36	SSLWSEYHDDFR FYPAFIHILAR TTVDISQWHR	26	5.9
6201	Methionine aminopeptidase	AMPM_ECOLI	13	59	No	29	5.6
6502	Adenylosuccinate lyase	PUR8_ECOLI	15	33	No	52	5.7
6603	Pyruvate kinase I	KPYK1_ECOLI	16	43	No	51	5.8

6703	DNA gyrase subunit B	GYRB_ECO57	12	24	No	90	5.7
7202	Dihydrodipicolinate synthase	DAPA_ECOLI	9	42	No	31	6.0
7203	Phospho-2-dehydro-3-deoxyheptonate aldolase, Phe-sensitive	AROG_ECOLI	12	43	SITDACIGWEDTDALLR	38	6.1
7203	Glyceraldehyde-3-phosphate dehydrogenase A	G3P1_ECOLI	8	37	VPTPNVSVVDLTVR<br>LVSWYDNETGYSNK	36	6.6
7303	L-threonine 3-dehydrogenase	TDH_ECOLI	12	43	NVVITDVNEYR<br>MAALIQSGLDLSPIITHR	37	5.9
7303	Phenylalanyl-tRNA synthetase alpha chain	SYFA_ECOLI	7	28	NFFEEDLQIR<br>ADHDTFWFDTTR	37	5.8
7306	Lactose operon repressor	LACI_ECOLI	11	41	No	39	6.4
7401	Carbamoyl-phosphate synthase small chain	CARA_ECOLI	11	48	No	41	5.9
7402	Cysteine desulfurase	ISCS_ECOLI	15	38	EGFEVTYLAPQR<br>SGTLPVHQIVGMGEAYR	45	5.9
7402	Protein tolB	TOLB_ECOLI	4	12	VSDYDGYNQFVVHR<br>IAYVVQTNGGQFPYELR	46	7.0
7501	D-3-phosphoglycerate dehydrogenase	SERA_ECOLI	11	29	GIPVFNAPFSNTR<br>YSDNGSTLSAVNFPEVSLPLHGGR	44	6.0

7501	Serine hydroxymethyltransferase	GLYA_ECOLI	9	24	AMVEVFLER VRQEEHIELIASENYTSPR	45	5.9
7501	Queuine tRNA-ribosyltransferase	TGT_ECOLI	5	16	ILEHVCPQIPADKPR GPILTDSGGFQVFSLGDIR	43	6.0
7507	Bifunctional protein glmU	GLMU_ECOLI	14	33	No	49	6.1
7603	ATP synthase subunit alpha	ATPA_ECOLI	20	42	No	55	5.8
7609	Fumarate hydratase class I, aerobic	FUMA_ECOLI	13	31	No	60	6.1
7610	Anthranilate synthase component II	TRPG_ECOLI	23	56	No	57	6.0
7704	ATP-dependent protease La	LON_ECOL6	14	17	HIEINGDNLHDYLGVQR	87	6.0
8201	Succinyl-CoA ligase [ADP-forming] subunit alpha	SUCD_ECOLI	10	47	No	30	6.3
8202	Tryptophanyl-tRNA synthetase	SYW_ECOLI	17	56	SGAQPSGELTIGNYMGALR	37	6.3
8205	Glyceraldehyde-3-phosphate dehydrogenase A	G3P1_ECOLI	17	58	No	36	6.6
8302	Glyceraldehyde-3-phosphate dehydrogenase A	G3P1_ECOLI	14	50	No	36	6.6
8302	HTH-type transcriptional repressor purR	PURR_ECOLI	8	29	EIGVIPGPLER VPQDVSLIGYDNVR	38	6.3

8306	Lactose operon repressor	LACI_ECOLI	11	41	No	39	6.4
8307	Protein sopB	SOPB_ECO57	10	50	No	35	7.7
8307	Lipoyl synthase	LIPA_ECO24	11	43	No	36	8.1
8402	Nicotinate phosphoribosyltransfer ase	PNCB_ECOLI	19	49	No	46	6.1
8403	Erythronate-4-phosphate dehydrogenase	PDXB_ECOLI	17	50	No	41	6.2
8403	Lactose operon repressor	LACI_ECOLI	16	57	No	39	6.4
8501	Citrate synthase	CISY_ECOLI	9	24	YSIGQPFVYPR ITFIDGDEGILLHR	48	6.2
8505	Transcription termination factor Rho	RHO_ECOLI	16	44	DVIILLDSITR ILFENLTPLHANSR	47	6.8
8505	Biotin carboxylase	ACCC_ECOLI	10	25	FHAPGGFGVR HVEIQVLADGQGNAIYLAER	49	6.7
9601	Aerobic glycerol-3-phosphate dehydrogenase	GLPD_ECOLI	24	50	No	57	7.0
9601	Sulfite reductase [NADPH] hemoprotein beta-component	CYSI_ECO24	15	33	No	64	7.3

0202/0204	Spermidine/putrescine-binding periplasmic protein	POTD_ECOLI	9	31	No	39	5.2
2904A	Carbamoyl-phosphate synthase large chain	CARB_ECOLI	17	22	No	118	5.2
2904B	Phosphoribosylformylglycinamidine synthase	PUR4_ECOLI	19	21	No	141	5.2
